# Association of stigmatizing attitudes with people’s opinion of depression as a valid reason for sickness absence: A Swedish vignette study

**DOI:** 10.3233/WOR-205181

**Published:** 2022-10-17

**Authors:** Monica Bertilsson, Jesper Löve, Johan Martinsson, Lena Wängnerud, Gunnel Hensing

**Affiliations:** a Department of Public Health and Community Medicine, The Institute of Medicine, University of Gothenburg, Gothenburg, Sweden; b Department of Political Science, University of Gothenburg, Gothenburg, Sweden

**Keywords:** Stigma, return-to-work, public health, vignette study

## Abstract

**BACKGROUND:**

Depression is a common cause of sickness absence (SA) and also highly associated with stigma. Few studies have addressed the role of stigma in relation to SA.

**OBJECTIVE:**

To investigate if attitudes to depression were associated with the public’s opinion of depression as a valid reason of SA.

**METHODS:**

The study population (*n* = 2413) originated from a web-based panel of citizens. The survey included a short vignette describing a person with symptoms of depression and the person’s work tasks, followed by a question on recommendation of SA. Negative attitudes were measured by the Depression Stigma Scale. Logistic regressions were used to estimate the odds ratios (OR) for the likelihood of not recommending SA, controlling for individual and work-related co-variates.

**RESULTS:**

The crude association between negative attitudes and not recommending SA was OR 2.15 (95% CI, 1.76–2.62). In the fully adjusted model the OR was 1.76 (95% CI, 1.40 –2.21) for not recommending SA.

**CONCLUSIONS:**

Participants with negative attitudes to depression were more likely to not consider depression as a valid reason of sickness absence. The study supports theories on layered stigma; attitudes from one arena are related to other arenas. Future studies are needed to confirm our findings.

## 1 Introduction

Depression disorders are common in the general population but are still associated with negative attitudes and carry stigma [1–4]. General population-based studies have reported that 17% –35% of people agreed that “it is best to avoid people with depression” [5, 6]. Studies have also found links between political ideology and stigma to depression, where people with a political right-wing ideology have more negative attitudes towards depression than people with a left-wing ideology [7, 8]. Depression is also a main reason for long-term sickness absence and research have pointed out that mental health stigma needs to be addressed in sickness absence and return to work (RTW) processes since it is likely to interfere with RTW and complicate work participation [2–4, 9, 10]. A sample of nurses found higher prevalence of negative attitudes towards colleagues returning to work after sickness absence due to common mental disorders (CMD) compared to returning from physical disorders [11]. In addition, employees tend to have a more negative attitude against co-workers with CMD compared with co-workers having physical disorders [2, 12]. In this study we focus instead on the association between stigmatizing attitudes to depression and the public’s view of depression as a valid reason for sickness absence. If an association is found it is an imperative to deal with layered stigma; depression attitudes might hamper employees to take a needed time off work while depressed and a possible deterioration of their illness [13–15].

Shame, psychological stress and decreased self-esteem have been described as effects of stigma [16]. Swedish studies have found that a higher proportion of persons sick-listed with CMD felt ashamed for being off sick compared to those sick-listed for other reasons [17, 18]. This indicates that some disorders might be considered more legitimate for sickness absence than others. Of importance is that Knapstad et al. found that the sickness absence duration was longer among those feeling ashamed [18]. Other findings show that employees with depression avoid both going on sick leave and tell about their depression at work of fear for stigmatization [1, 13]. Workers with comorbid conditions, once back at work, they find it more difficult to disclose depression than cancer or heart diseases [9]. However, if employees do not disclose their ailments due to real or imagined negative attitudes from the manager and co-workers it will be difficult to do adjustments in work tasks and demands. Thus, rehabilitation efforts and interventions might be misguided and of no or limited effect [3, 4, 19].

The departure of this study was to investigate layered stigma, that is, if stigma from one arena (public negative attitudes towards depression) might be associated with other arenas (sickness absence and rehabilitation) [15]. More specifically, the aim was to estimate the association between depression attitudes and depression as a valid reason for sickness absence. The hypothesis was that individuals with negative attitudes to depression would be more restrictive in recommending sickness absence.

## 2 Methods

### 2.1 Design and setting

This study is part of the New Ways –Mental Health at Work research program, aimed at identifying, treating and supporting persons with CMD to remain in work. To test the hypothesis we used an experimental vignette design with data-collection through a web-survey. The web-survey was distributed to a self-recruited panel of the Swedish public in December 2014. We developed survey questions and a short case vignette that were nested in the 13th survey to the Citizen Panel at the Laboratory of Opinion Research (LORE) at University of Gothenburg [20]. The Citizen Panel has ethical approval from the Regional Ethical Review Board in Gothenburg, Sweden (Dnr: 189-14).

### 2.2 Study population

**Fig. 1:**
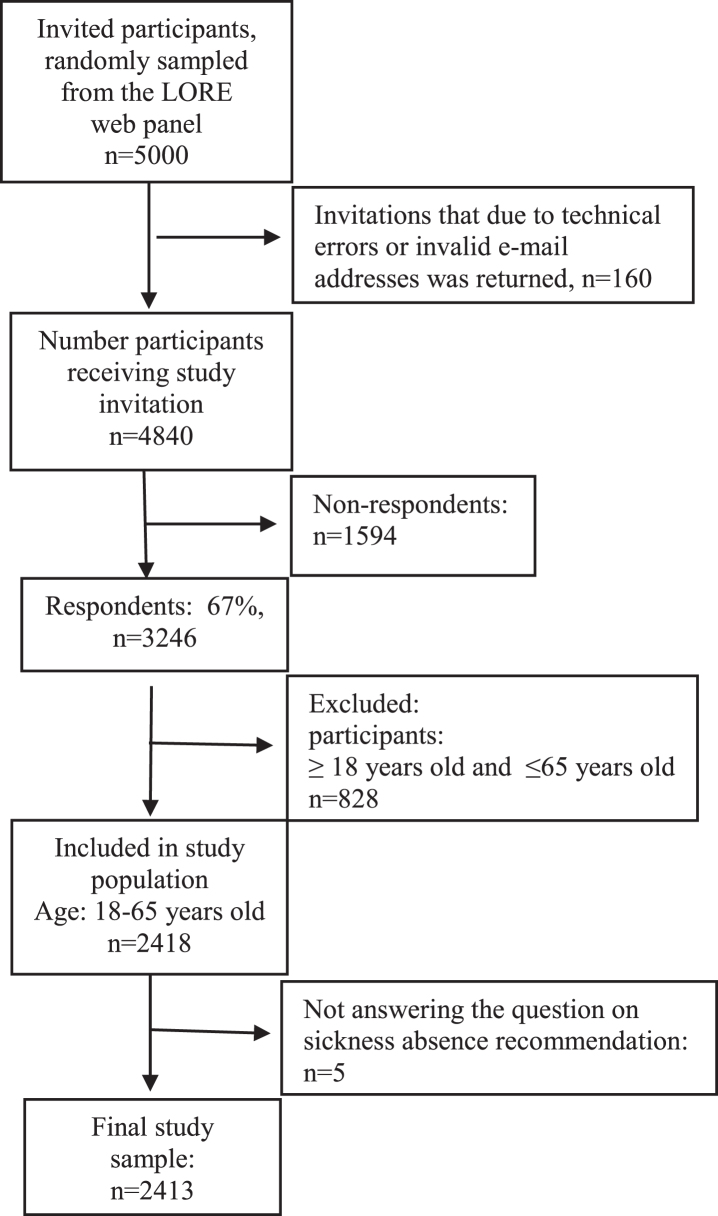
Flowchart over invited participants from the LORE web-panel to study population.

Five thousand individuals were invited to participate. Two reminders were sent to those who did not respond. The overall response rate was 67% (*n* = 3246, Fig. 1). We included individuals aged, 18–65 years (*n* = 2418) since the research question was most relevant for those in working ages. We excluded those who did not answer the question on sickness absence recommendation (*n* = 5), resulting in a final study sample of *n* = 2413 (Fig. 1).

### 2.3 Negative attitudes towards depression

Negative attitudes towards depression were measured by the Depression Stigma Scale (DSS), used in several other studies [21, 22]. The scale was translated into Swedish by the research group. The DSS consists of two subscales; we used the DSS-Personal stigma sub scale which measures the participants’ personal attitudes towards depression (public stigma). The scale is a 9-item subscale (score range, 9–45); higher scores on the DSS represent a greater level of stigma. The scale was created as the sum of nine statements with a five-point (Likert) response scale ranging from “strongly disagree” (1) to “strongly agree” (5). Only individuals with a value for all items received a score on the final scale, thus, 31 individuals (ie, 1% of the original study sample) were excluded.

The scale was dichotomized at the 3rd quartile (score, 19) into having no negative attitudes (score≤19) and as having negative attitudes (score≥20). This enabled the participants with the most negative attitudes to be separated from the others.

The Swedish version of the DSS questionnaire has been found to have acceptable internal consistency; i.e., Cronbach’s alpha = 0.78. However, we did find potential floor effects (i.e., low mean values) in four of the DSS items (depression is not a real medical illness; people with depression are dangerous; it is best to avoid people with depression so you don’t become depressed yourself; people with depression are unpredictable), reducing the response variation. The other five items in the DSS scale were: people with depression could snap out of it, if they wanted; depression is a sign of personal weakness; if I had depression, I would not tell anyone; I would not employ someone if I knew they had been depressed; I would not vote for a politician if I knew they had been depressed.

### 2.4 Recommendations of sickness absence

**Fig. 2:**
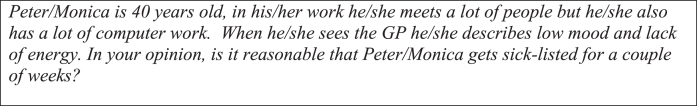
The case vignette in the web-panel questionnaire.

For this study, we developed a written case-vignette briefly describing a person’s work tasks and common symptoms of mild to moderate depression (Fig. 2). The vignette was based in earlier research in our research group where we have interviewed workers with CMD about their capacity to work [14] and about early signs of work instability [23]. Mental and inter-personal work tasks have been found to be very troublesome for individuals with depression [14, 23, 24], therefore we choose to include descriptions of these types of work tasks in the vignette. The vignette formulations were discussed with researchers from different disciplines, including a senior Psychiatrist on how to capture symptoms of depression. Due to the web format the vignette had to be short, however the length is almost similar to other vignettes depicting mild to moderate depression in a work place context [25, 26]. The vignette design was not pre-tested because LORE have huge experience of using vignettes in the Citizen panel. The vignette was placed well before the DSS scale in the survey. After reading the vignette, the respondents were given a question on whether they thought that the person described in the vignette should be sick-listed or not, with the following response alternatives: yes absolutely, yes probably, probably not, absolutely not. The outcome variable was dichotomized into yes, recommending sickness absence (yes absolutely, yes probably) and no, not recommending sickness absence (probably not, absolutely not). To avoid potential gender effects, half of the participants were randomly shown a vignette about Peter (male gender); the other half were shown a vignette about Monica (female gender).

### 2.5 Co-variates

The lack of social context in stigma studies have been criticized in a recent review because this might have an impact on attitudes [15]. We have chosen to include co-variates related to negative attitudes to CMD, but since sickness absence also is subjected to attitudes, we included co-variates related to attitudes of sickness absence as well: gender, age, education, political ideology, self-rated health and self-reported sickness absence [27–29].

Political ideology was measured through the question “People sometimes talk about political opinions on a left-right ideological scale. Where would you place yourself on such a left-right ideological scale?”. The response alternatives ranged from “far to the left” (0) to “far to the right” (10) with the midpoint (5) labelled “neither left nor right”. The 11-point scale was categorized into left-wing ideology (0–3), middle position (4–6), right-wing ideology (7–10). Self-reported left-right ideology is widely used within political science and the 11-point scale used in this study has been evaluated and found to perform well as a measurement instrument [30].

As work place factors we used work sector (state, community, regional, private and non-profit organizations), and work position categorized to managers (higher managers, managers, first-line managers), self-employed without employees and co-workers (white collar and blue collar employees).

We used five individual co-variates. Self-rated health was measured by the question “how do you rate your general health” with five response alternatives (very good health, fairly good health, neither good nor bad health, fairly bad health, very bad health), and self-reported sickness absence in the last 12 months (none, 1–7 days, 8–30 days, 1–3 month, 4–12 month). In addition, sex (woman, man, other), age and educational level (primary, secondary, upper secondary, higher, PhD degree) were analysed.

### 2.6 Statistical analyses

General analyses of statistical power to be able to perform sub-group analyses was conducted before the data collection [20]. A specific power analysis for the current study was also performed but only to reassure that we would include a sufficient number of both women and men.

To estimate the stigma score we calculated the range and mean score. Co-variates significantly (chi-square test, *p* < 0.05) associated with the outcome were included in the regression analysis. Multicollinearity (> 0.60) was checked among the co-variates using Person correlation, and original variables without dichotomization were used. Logistic regression analyses were used to calculate crude and adjusted odds ratios (ORs) with 95% confidence intervals (95% CIs) for the probability of not recommending sickness absence in the case vignette with regard to the independent variable. Recommending (i.e., “yes”) sickness absence was used as the reference category. Of the co-variates, age was entered as a continuous variable; all other co-variates were categorical and used as described above. In the multivariable logistic regression analyses, all models were adjusted for sex and age. In addition, we adjusted for education in model 2, self-rated health and self-reported sickness absence in model 3, work sector and work position in model 4, and political ideology in model 5. We included all co-variates in the final model. To test for a possible effect of being in employment or not, the final model was rerun after exclusion of participants not currently in paid work (unemployed, students, pensioners, other labour market situation) (*n* = 496). IBM SPSS statistics 22 was used in the analyses (IBM Corp. Armonk, NY, 2011).

## 3 Results

### 3.1 Demographic details

The study population’s demographic details are shown in Table 1. Compared with the overall Swedish population, people with a university education were overrepresented and young people were underrepresented. In the multicollinearity test among co-variates, the largest correlation was found between self-rated health and sickness absence (*r* = 0.33), statistically significant at *p* = 0.01.

**Table 1:** Study population characteristics

	Proportions (%)	Frequency^*(*n*=)^
Sex
Women	48	1156
Men	51	1239
Other	1	13
Age
18–29	10.5	253
30–39	18.6	448
40–49	22.4	541
50–59	27.0	652
60–65	21.5	519
Education
Primary or less	3.3	80
Secondary	21.6	520
Upper secondary	11.4	275
Higher education	60.1	1147
Doctoral degree	3.5	85
Labor market situation
Working	79.4	1911
Unemployed	3.1	75
Student	5.6	156
Pensioner	6.5	134
Other	5.4	131
Work position
Managers	31.1	735
Self-employed without employees	5.0	118
White collar employee	43.2	1020
Blue collar employee	20.7	488
Work sector
State	12.1	281
Community	21.4	497
Regional	8.0	185
Private	54.9	1276
Non-profite organisations	3.7	87
Political ideology
Left wing	32	774
Middle position	36	855
Right-wing	32	782
Self-rated health
Good health (good, fairly good)	76.1	1830
Neither good or bad health	16.0	385
Bad health (fairly, very bad)	7.9	191
Self-rated sickness absence last 12 month
No sickness absence	64.6	1553
1–7 days of sickness absence	23.7	569
8–30 days	5.5	133
1–3 month	2.2	52
4–12 month	4.0	97

^*^numbers not adding up to 2413 is due to internal missing.

In this study population the stigma scores ranged from 9 to 45. The mean stigma score was 15.89 (standard deviation [SD] 5.5). Among these, 77% scored≤19 points for the DSS and were classified as *not* having negative attitudes to depression; 23% scored 20–45 points and were classified as having negative attitudes. Of those who had negative attitudes to depression 34% recommended in favor and 66% against sickness absence; the corresponding figures in the group who did not have negative attitudes were 52% in favor and 48% against.

### 3.2 The likelihood of not recommending sickness absence for the case vignette

The crude OR was 2.15 (95% CI, 1.76–2.62) for the likelihood of *not* recommending sickness absence among individuals with negative attitudes to depression compared with those with no negative attitudes (Table 2). The odds ratio was only slightly changed in models 1–3, adjusted for individual level co-variates (age, gender, education, self-rated health, self-reported sickness absence). Adjusting for work place factors (work sector, work position) in model 4 did not change the OR. Adjusting for political ideology attenuated the OR to 1.64 (95% CI, 1.33–2.02). In the fully adjusted model, negative attitudes to depression were still associated with *not* recommending sickness absence (OR, 1.76; 95% CI, 1.40–2.21). The sensitivity analysis excluding the individuals in the study population who were not employed (*n* = 496) did not change the final result (OR 1.78; 95% CI, 1.38–2.29).

**Table 2:** Crude and adjusted odds ratios (OR) with 95% confidence interval (CI) for the likelihood of not recommending sickness absence in a written case vignette of a depressed person by negative attitudes to depression (*n* = 2413, 52% men and 48% women)

	N (exposed)	%	Crude OR 95% CI	Model 1 OR 95% CI	Model 2 OR 95% CI	Model 3 OR 95% CI	Model 4 OR 95% CI	Model 5 OR 95% CI	Model 6 OR 95% CI
No negative attitudes	874	48.0	1	1	1	1	1	1	1
Having negative attitudes	371	66.0	2.15	2.04	2.16	2.03	2.15	1.64	1.76
			(1.76–2.62)	(1.67–2.50)	(1.76–2.65)	(1.65–2.49)	(1.74–2.66)	(1.33–2.02)	(1.40–2.21)

Model 1: adjusted for gender and age. Model 2: adjusted for gender, age, education. Model 3: adjusted for gender, age, self-rated health, sickness absence the last 12 month. Model 4: adjusted for gender, age, work sector, work position. Model 5: adjusted for gender, age, political ideology. Model 6: adjusted for gender, age, education, self-rated health, sickness absence the last 12 month, work sector, work position, and political ideology.

## 4 Discussion

In line with our hypothesis we found that, negative attitudes to depression was associated with *not* recommending sickness absence, and this was true also in the fully adjusted model.

### 4.1 Interpretation of the findings

This study is the first of its kind. Earlier studies have either studied negative attitudes to depression or to sickness absence separately, and an association with gender, age and education was found [27, 28, 31, 32]. However, adjusting for these factors did not attenuate the OR for not recommending sickness absence. Neither did self-rated health and own experience of sickness absence contribute to the explanation [29]. Further, work place factors did not have an effect on the OR [28, 32].This was intriguing, particularly because 55% of the participants worked in the private sector and earlier research have found people in private sector to have more negative attitudes to depression [32].

In this study, political ideology had the strongest attenuating effect. Studies from political sciences have found that right-wing sympathizers have more negative attitudes to depression and restrictiveness towards welfare solutions such as sickness absence [7, 33]. Most commonly, these patterns are explained by personality factors such as “the closed authoritarian personality syndrome”. This means that stigmatizing attitudes are seen as a response to a psychological need for routines and predictability, which, it is argued, is offered to a higher degree in conservative ideology than in liberal or left-wing ideologies [7, 34]. More recently, however, research has started to distinguish between different forms of conservative ideologies in discussions on authoritarianism and related stigmatizing attitudes. Conservatives may hold more negative attitudes to depression based on a worldview where personal agency and responsibility are highly valued and therefore find sick-leave less acceptable, or based on a worldview where depression is seen as a threat that has to be controlled. It is plausible that our indicator on political ideology, the left-right scale, captures effects of both forms of conservatism [35].

A plausible explanation for the main finding can be found in Krane et al.’s [12] qualitative Nordic study, in which the researchers identified a category they called “acceptable causes of sickness absence”. In Krane et al’s study mental health disorders were perceived as a “grey zone” where the non-visibility of both symptoms and decreased work capacity contributed to doubts on whether these illnesses were acceptable causes for sickness absence or not [12]. Added to this, population-based studies have found that between 35% and 58% of the respondents perceived people with depression as weak [5, 6, 36]. Similarly, a Finnish survey investigated stigma conceptualized as “depression as a matter of will”, as many as 58% answered that people with depression “should pull themselves together” and 41% viewed mental problems as a sign of weakness [36]. Such perceptions and attitudes are likely to add to the notion that depression is not a valid reason for sickness absence in people who have negative attitudes to depression. Other plausible explanations to the main finding in this study (residual confounding) should be investigated in further studies. Examples are experiences of employees/workmates with CMDs, the particular relationship (close/distant) of these employees/workmates, and possible own experience of depressive symptoms.

Angermeyer et al. [15] argue that public stigma, on a collective level, forms a cultural context altering peoples’ everyday practice affecting people within that context. In work places, it is plausible to believe that the collective level of negative attitudes affects the psycho-social work environment negatively in relation to mental ill-health and subsequent sickness absence. Disclosure, support and work adjustments are most probably hampered in such settings [1, 19]. Among co-workers, an OECD panel from Denmark found that as many as 55% hesitated about working with someone with CMD, compared with 10% if the colleague used a wheel-chair [2]. Findings from qualitative studies show that depression is considered taboo to talk about at work [13, 37] and in a large survey among Swedish managers, it was less likely that managers with negative attitudes to depression was informed about the employees’ CMD through self-disclosure, compared with managers with no negative attitudes [38]. These findings points to the importance of the cultural context in work places. To address negative attitudes in work places thus could be one way to open up for higher transparency and communication in sickness absence processes. Lecours et al. identified the theme “acting for the mental health of others”, as one important activity among workers themselves towards a better psycho-social work environment to prevent CMD, which among others included the action to “openly speak about mental health to normalize the subject” [39]. Among Union representatives, it was found and argued that work environments sensitive to mental health issues and without prejudices were highly important in RTW-processes [40]. Managers, as possible key actors in such work should be targeted more directly in future studies. Improved knowledge on CMDs as such and their possible effects on managers’ preventive strategies [41] and employees’ work capacity can be a first step, but more direct work with changing attitudes and norms is also needed [42, 43].

In general, stigma carries negative consequences for individuals [1, 2, 19]. However, in relation to sickness absence which is described as a phenomenon and decision influenced by several factors [13, 44], negative attitudes to depression might constitute a (supportive) barrier for sick-leave and thus maintain individuals to stay at work. This could be seen as a favorable effect reducing the risk of long time absence. Put under hard pressure at work sick leave might be seen as the sole solution for an individual with depression, and barriers to sickness absence could promote decision-making about other solutions that might be more significant, such as adaptation or change in the work tasks. Although stigma can theoretically be thought of as a barrier to sickness absence, it has though mainly shown negative influences on individuals’ lives and several studies and reviews emphasize the need for better understanding of stigma to CMD as a potential barrier to work participation [3, 4, 10, 19]. Therefore, reduction in negative attitudes to depression through increased knowledge among the public and in work places is one way forward to achieve better adaptation at work and support for persons with depression and other mental health problems [2–4, 43].

The findings from this first study on the association between negative attitudes and vignette-based recommendations of sickness absence goes in the same direction as other stigma studies which have found layered stigma to e.g. care-seeking and job-opportunities among persons with mental ill-health [42]. Still, the current finding contributes to our understanding of how attitudes and norms co-vary and needs to be considered in the complex interrelationship between work, depression and sickness absence. Put more explicit, our findings indicate that stigmatizing attitudes to depression cannot be separated from how sickness absence with depression should be approached and dealt with professionally. The relation between attitudes and actions taken (or not taken) among stakeholders and even the sick employees themselves, might exacerbate a distressing situation, or avoid one [9, 10, 13]. Still, the current findings need to be replicated in future studies on written or video vignettes in Sweden and other countries as well.

### 4.2 Practical implications

Stigma needs to be addressed in society but interventions might be more successful if specific groups are targeted [42, 43]. Corrigan et al. [42] suggest employers and health care providers, significant groups in relation to sickness absence and RTW. To speed up return to work processes (RTW), several reviews have concluded that stigma to CMD needs to be better understood and addressed [3, 4, 19]. These reviews emphasize the importance to counteract stigma in the work context, and urged employers and work places and the health care to take actions to minimize the effects of stigma. However, another problematic situation that needs attention, is negative attitudes to mental health problems among health care personnel themselves [10, 11, 45], which might both contribute to and reinforce negative attitudes to CMD. Positively, Henderson et al. [45] found some evidence that education about mental health decreases such negative attitudes in health care personnel.

A further important matter is the public stigma’s effect on the individual’s view of him/herself, leading to self-stigma [42, 46]. In people with depression, self-stigma has been associated with decreased help-seeking and avoidance of telling about their situation [22, 46]. Other studies showed that affected persons hesitates to disclose their problems at work and to take necessary sick-leave, due to fear of stigma [1, 13]. Sadly such behaviors are most likely to hamper adequate interventions both in health care and at work. Contrary to expected, however, Munir et al. [47] found that employees albeit telling about their CMD to their employers were less likely to receive work adjustments compared to employees with somatic conditions. Similar result was found by Telwatte et al. [48] in a vignette study to employers and HR personnel, where employees in vignettes with CMD related work impairment were least likely to be granted work adjustments. It is urgent that both work organizations and health care develop routines and preparedness for actions in how to address counterproductive negative attitudes.

### 4.3 Strengths and limitations

The major strengths of this study was the use of a validated stigma instrument developed for depression to use in general population based samples. The inclusion of contextual co-variates was important and follows suggestions from earlier research [15]. The chosen co-variates were all associated with both depression and sickness absence. The vignette was developed for this specific study and based in earlier qualitative research from our group [14, 23]. To increase validity, the vignette formulations were discussed and revised. That the vignette not only described symptoms but also specified work tasks was a strength since both add vital information in relation to sickness absence. To use symptoms related to mild/moderate depression might have been too vague. However, according to the Swedish National Insurance Medicine Decision Support (for physicians and Social insurance agency officials) the recommended sickness absence duration for mild depression is three month and six month for moderate to severe depression. Further, we used a female and a male vignette to manage gender bias [49, 50]. We achieved a high response rate and since only participants of working age were included, the external validity was strengthened.

Limitations were mainly related to measurements. We have a potential floor effect in some of the DSS items. This reduced variation in some items might be due to the fact that DSS was developed in another cultural context while the specific items either were understood in a more negative way in Swedish or that the respondents in fact did not find them relevant. Due to the high proportion with university education these questions might be answered in a socially desired way [15]. The floor effect might have contributed to a lower proportion of respondents reporting negative attitudes to depression. The sample had a larger proportion of highly educated individuals which most likely has introduced a lower proportion of negative attitudes which mainly influence the estimates of proportions. The educational level was adjusted for in the regression analyses and thus, did not influence estimates of associations.

The study was performed with a short and easy to read vignette of importance in web surveys. Thus, the development of the vignette was an act of balance between brief and easy, and representing the complex nature of depressive symptoms and work tasks. The vignette might have been too general, and trigger negative attitudes to sickness absence rather than attitudes to depression. In retrospect a way to get more information in this could have been to include a similar vignette representing for example musculoskeletal disorders distributed to half of the study sample. This, and also the use of video vignettes are recommendations for future studies [51]. To enhance external validity participants were not taken from their daily habitat to an experimental environment but reading the vignettes and filling in the questionnaires from home [52]. The participants also represent a community sample and not a selected/specific group [52]. Even though a high response rate the self-recruited sample restricts the generalizability of the study. Still, this potential bias was potentially reduced in the adjusted analyses. To minimize response bias, the vignettes were given names (Monica/Peter) [52]. It should also be recognized that sickness absence is a phenomenon subjected to attitudes in itself [27–29] which might have distorted the result.

## 5 Conclusions

This first study on the association between attitudes to depression and recommendations of sickness absence found an association between negative attitudes to depression and *not* recommending sickness absence. The association remained also after adjustment for several individual and work related contextual factors. The study supports theories on layered stigma; attitudes from one arena are related to other arenas. Future studies are needed to confirm our findings.
